# AlN/GaN Digital Alloy for Mid- and Deep-Ultraviolet Optoelectronics

**DOI:** 10.1038/s41598-017-12125-9

**Published:** 2017-09-19

**Authors:** Wei Sun, Chee-Keong Tan, Nelson Tansu

**Affiliations:** 1 Center for Photonics and Nanoelectronics, Department of Electrical and Computer Engineering, Lehigh University, Bethlehem, PA 18015 USA; 2 Department of Electrical and Computer Engineering, Clarkson University, Potsdam, NY 13699 USA

## Abstract

The AlN/GaN digital alloy (DA) is a superlattice-like nanostructure formed by stacking ultra-thin ( ≤ 4 monolayers) AlN barriers and GaN wells periodically. Here we performed a comprehensive study on the electronics and optoelectronics properties of the AlN/GaN DA for mid- and deep-ultraviolet (UV) applications. Our numerical analysis indicates significant miniband engineering in the AlN/GaN DA by tuning the thicknesses of AlN barriers and GaN wells, so that the effective energy gap can be engineered from ~3.97 eV to ~5.24 eV. The band structure calculation also shows that the valence subbands of the AlN/GaN DA is properly rearranged leading to the heavy-hole (HH) miniband being the top valence subband, which results in the desired transverse-electric polarized emission. Furthermore, our study reveals that the electron-hole wavefunction overlaps in the AlN/GaN DA structure can be remarkably enhanced up to 97% showing the great potential of improving the internal quantum efficiency for mid- and deep-UV device application. In addition, the optical absorption properties of the AlN/GaN DA are analyzed with wide spectral coverage and spectral tunability in mid- and deep-UV regime. Our findings suggest the potential of implementing the AlN/GaN DA as a promising active region design for high efficiency mid- and deep-UV device applications.

## Introduction

III-Nitride semiconductor materials have been commonly recognized as one of the key promising material platform in optoelectronic applications during the past decades^1–10^. The material epitaxy techniques and active region design have been developing rapidly for solid-state lighting^11–16^, and solar energy harvesting^17–22^. The implementation of the narrow band gap InGaN ternary alloy into GaN-based active region leads to the unprecedented progress of high efficiency light emitting diode (LED) and laser diode (LD) and solar cell techniques in visible and longer wavelength applications^11–22^. On the other hand, utilizing the wide band gap AlN-based materials also enables the emerging development of III-nitride ultraviolet (UV) emitters for material sterilization and water purification applications^23–44^. Specifically, The AlGaN material has the capability of tuning its band gap from 3.437 eV (GaN) to 6 eV (AlN) by changing the Al-content in the ternary compositional alloy^10^. Integrated with AlN, the AlGaN based active region has shown its potential in covering mid- and deep-UV spectral regime from 210 to 360 nm for device applications.

Extensive studies have been conducted on the AlGaN based quantum well (QW) active region for mid- and deep-UV device applications^23–44^. However, the conventional high Al-content AlGaN based deep-UV devices still experience the low quantum efficiency issues attributed to several aspects, such as poor material quality^33,34^, difficulty in achieving sufficiently high p-type doping in material^34,35^, charge separation^36^, and the valence band crossover issue^37–40^. Unlike the InGaN material system, the top valence subband of the AlGaN based QW will switch from the heavy hole (HH) band to the crystal-field split-off hole (CH) band at higher Al-content (>57% in bulk AlGaN)^37–40^. Such phenomenon eventually results in the undesired dominant transverse-magnetic (TM) polarized emission instead of the desired dominant transverse-electric (TE) polarized emission^37–40^. Several strategies have been proposed to reverse the valence band crossover issue by engineering the strain and barrier composition^41^, increasing the well thickness or injected carrier density^42^, and inserting delta-GaN layer into the AlGaN QW^43,44^. Additionally, recent studies have been carried out on the new type of wide bandgap material AlInN and its quantum structure targeting deep-UV device applications^45–47^. However, the material epitaxy of high quality AlInN alloy and its experimental integration into quantum nanostructure for deep-UV emitter application are still at early stage^45–47^. Thus, new strategies to achieve high efficiency AlGaN-based active region are still required for deep-UV device applications. In addition, various 2-dimensional (2D) materials and their nanostructures have also been widely employed for optoelectronic applications with remarkable advantages^48,49^. However, fabricating 2D devices in large scale and integrating them with mature III-Nitride-based electronic and optoelectronic devices are still at the early stage of development. Thus, having a novel active region design based on the mature III-Nitride material platform remains essential for superior devices integrations.

Recently, the short-period III-Nitride superlattice nanostructures have been targeted to achieve the comparable electronic and optoelectronic properties as the conventional III-Nitride ternary alloys. Several studies on the InN/GaN short-period superlattices and digital alloys (DAs) have been reported to obtain tunable optoelectronic properties at red and longer spectral regime^50–56^. The use of III-Nitride DA opens up the ability to tune the electronics and optoelectronics properties of new compounds – especially those which are hard to be grown (i.e. high In-content InGaN)^56^. Meanwhile, a series of studies have been conducted including the First-Principle calculation and experimental demonstration on the AlN/GaN superlattice-like heterostructure and monolayer (ML)-scale ultra-thin QW structures for deep-UV applications^57–63^. Those studies have shown the potential of using AlN/GaN superlattice and ultra-thin QW structures to overcome the fundamental issues of high Al-content AlGaN QW active region^58–63^. However, most of the prior-mentioned works mainly focused on the structure with relatively thick AlN barrier (>8 MLs). The effects of ultra-thin AlN barrier and GaN well layers on the electronic and optoelectronic properties in the AlN/GaN superlattice-based digital alloy structure still require significant clarifications.

In this work, we present a comprehensive study on the electronic and optoelectronic properties of the AlN/GaN DA consisting of ultra-thin (≤4 MLs) AlN barriers and GaN wells for mid- and deep-UV applications. The band structures and carrier wavefunctions of the AlN/GaN DA are evaluated numerically and compared to those of the ultra-thin GaN QW with AlN barriers. Specifically, the effective energy gap of the AlN/GaN DA are calculated as function of the thicknesses of the GaN wells and AlN barriers. Furthermore, the electron-hole wavefunction overlaps are also evaluated for the AlN/GaN DA nanostructure. In addition, we present the numerical assessment of the optical absorption properties of the AlN/GaN DA structures. Our evaluation on the AlN/GaN DA structures provides the insights on implementing AlN/GaN DA active region design for mid- and deep-UV device applications.

### Concepts and Modeling

**Figure 1:**
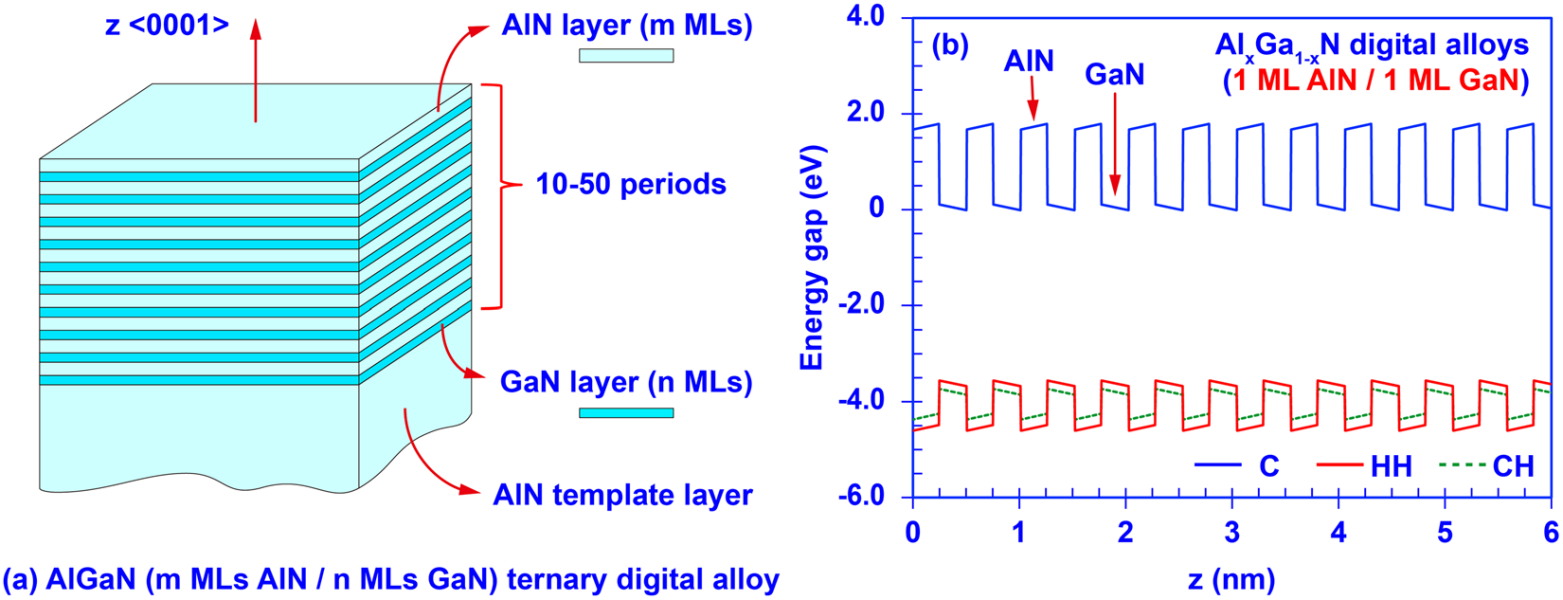
(**a**) Schematic illustration of the AlN/GaN DA formed by m MLs AlN and n MLs GaN ultra-thin binary layers; (**b**) Band diagram of the AlN/GaN DA formed by 1 ML AlN barriers and 1 ML GaN wells.

Similar to the InN/GaN DA nanostructure^56^, the AlN/GaN DA is an ultra-short period superlattice structure that can be formed by binary-periodic epitaxy of the AlN and GaN ultra-thin layers. Figure 1 shows the illustration of an AlN/GaN DA combined with its band line-up diagram. In our study, the thicknesses of the AlN barrier and GaN well regions within the AlN/GaN DA are presented by m and n MLs. Note that the thickness of the single ML III-Nitride material along the <0001> direction has to be one half of the lattice constant c of such III-Nitride material. Thus, the thickness of m MLs AlN barrier and n MLs GaN well is set to be *m* × 2.491 Å and *n* × 2.593 Å respectively^10^. In this study, the thicknesses of the AlN barrier and GaN well in the AlN/GaN DA are limited from 1 ML to 4 MLs. By using ultra-thin AlN and GaN layers, the carrier wavefunctions of the AlN/GaN DA are able to tunnel through the barrier regions and couple with one another. This phenomenon enables the inter-well resonant coupling effect that creates the minibands of the DA. Eventually, the miniband structures and the electronic / optoelectronic properties of the AlN/GaN DA can be engineered by tuning the thicknesses of the AlN barriers and GaN wells. In addition, utilizing the ultra-thin layers in the III-Nitride DA could also dramatically increase the electron-hole wavefunction overlap^56^, which in turn results in efficiency enhancement of the active region.

In this study, the energy band diagrams of the AlN/GaN DA are modeled based on a modified Kronig-Penney model including the strain effect induced by lattice mismatch and built-in polarization effect attributed to both spontaneous and piezoelectric polarization^64–68^. Figure 1(b) shows the band lineup of an AlN/GaN DA with 1 ML AlN barrier and 1 ML GaN well. Afterwards, the polarized energy band lineup is quantized by employing the finite differential method^68^. The miniband structures, quantum confined states, effective bandgap, and carrier wavefunctions of AlN/GaN DA are calculated by using the transfer matrix method^69^. In our analysis, the effective energy gap is defined as the energy difference between the ground confined energy states in conduction (C) and valence sub-bands. Lastly, the optical absorption spectra of the AlN/GaN were calculated applying the Fermi’s golden rule^64^ as shown in the equation (1):

1 $$a(\hslash \omega )={C}_{0}\sum _{n,m}{|{I}_{hm}^{en}|}^{2}\mathop{\mathop{\int }\limits^{{\rm{\infty }}}}\limits_{0}d{E}_{t}{\rho }_{r}^{2D}{|\hat{e}\bullet {{\boldsymbol{p}}}_{cv}|}^{2}L({E}_{hm}^{en}+{E}_{t}-\hslash \omega )\times [{f}_{v}^{m}({E}_{t})-{f}_{c}^{n}({E}_{t})]$$

where the ${{\rm{I}}}_{\mathrm{hm}}^{\mathrm{en}}$ represents wavefunction overlap integral, ${{\rm{\rho }}}_{{\rm{r}}}^{2{\rm{D}}}$ represents the two-dimensional joint density of states, and ${|\hat{{\rm{e}}}\bullet {{\bf{p}}}_{{\rm{c}}{\rm{v}}}|}^{2}$ is the momentum matrix element in the AlN/GaN DA. The $L({E}_{hm}^{en}+{E}_{t}-\hslash \omega )$ is the Lorentzian function for linewidth broadening, and ${f}_{v}^{m}({E}_{t})-{f}_{c}^{n}({E}_{t})$ shows the Fermi-Dirac population inversion factor. The material parameters used in this study were obtained from previous literatures^10,64,68^.

**Figure 2:**
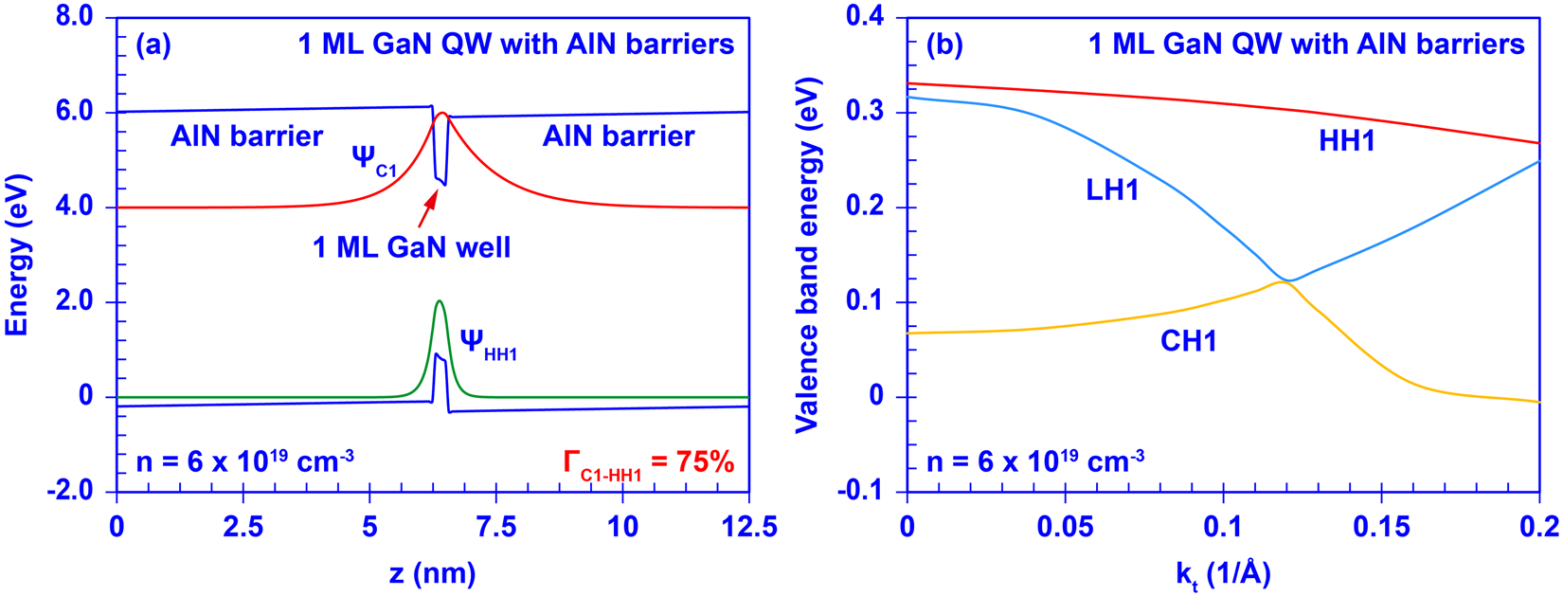
(**a**) Band diagram and carrier wavefunctions of the single ML GaN QW with AlN barriers; (**b**) valence band structure of the single ML GaN QW with AlN barriers.

Although the heavy-hole (HH), light-hole (LH), and the crystal-field split-off hole bands (CH) were all included in our calculation for the AlN/GaN DA structure, the valence band mixing effect was not considered in our transfer matrix method. In order to ensure the accuracy of our model and provide better comprehensive analysis, we also evaluate the electronic properties of the ultra-thin GaN QW with AlN barriers by employing the self-consistent 6-band ***k*** **·** ***p*** method^67,68^. The thickness of the GaN ultra-thin QW is varied from 1 ML to 4 MLs for comparison purpose. Figure 2(a) shows the energy band diagram along with the ground-state carrier wavefunctions in a 1 ML GaN QW with AlN barriers at carrier density n = 6 × 10^19^ cm^−3^. As shown in Fig. 2(a), by using the single ML GaN well, the wavefunctions of ground-state C band (C1) and HH band (HH1) are pushed to each other resulting in a significant enhancement of the electron-hole wavefunction overlap up to ~75%. Meanwhile, Fig. 2(b) shows that the valence subbands of the single ML GaN QW with AlN barriers are rearranged at the gamma point. The HH band becomes the top valence subband with sufficient energy separation of ~0.26 eV from the CH band. Our extensive study shows that the HH band will remain the top valence subband with increased energy separation from the CH band when the thickness of the GaN QW increases up to 4 MLs. Our 6-band ***k*** **·** ***p*** calculations and previous studies on the ultra-thin GaN QW with AlN barriers heterostructure have consistently shown the valence band rearrangement and the increase of the electron wavefunction overlap in the ultra-thin GaN QW with AlN barriers structure^59–63^.

## Results and Discussion

**Figure 3:**
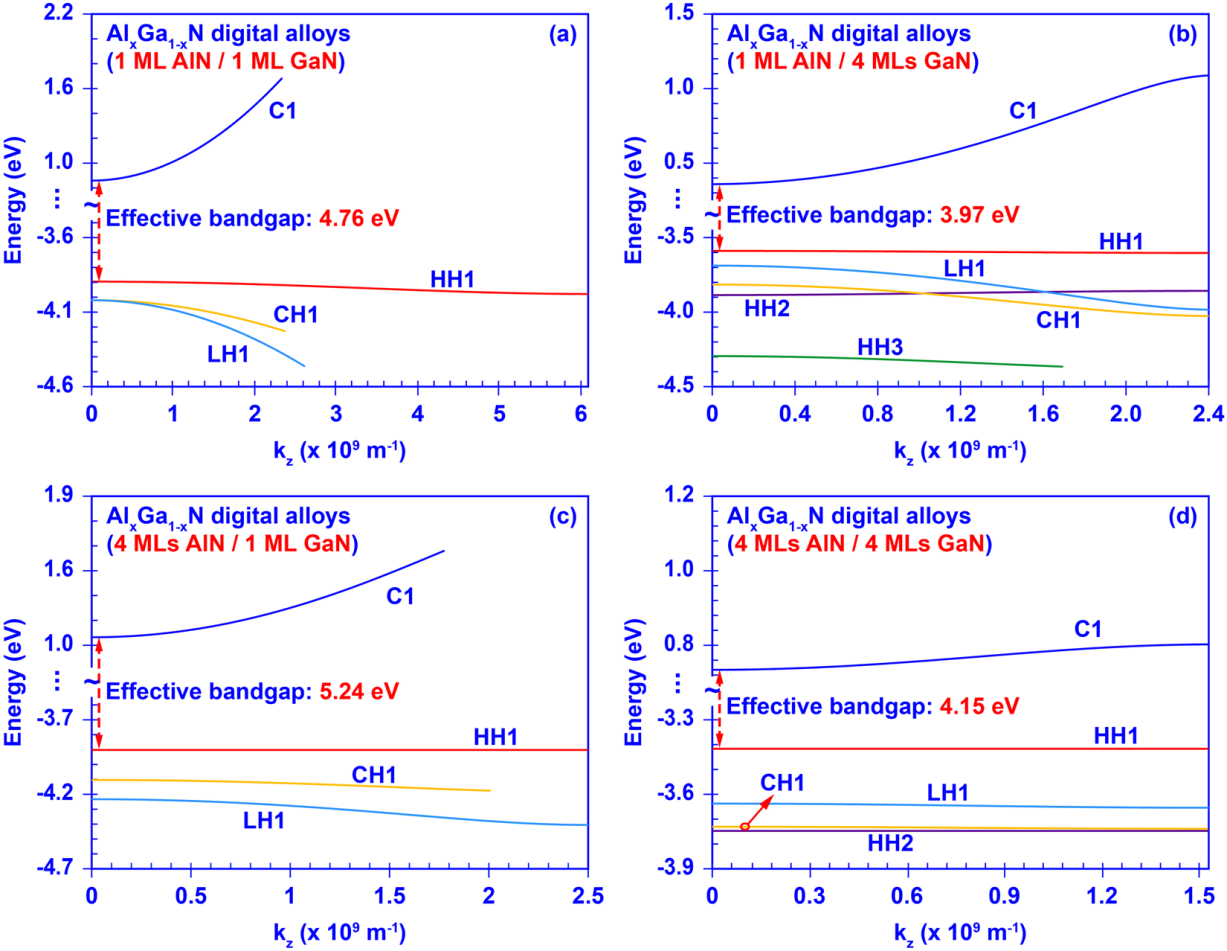
Miniband structures of the AlN/GaN DA formed by: (**a**) 1 ML AlN barriers with 1 ML GaN wells, (**b**) 1 ML AlN barriers with 4 MLs GaN wells, (**c**) 4 MLs AlN barriers with 1 ML GaN wells, and (**d**) 4 MLs AlN barriers with 4 MLs GaN wells.

Figure 3 shows the calculated miniband structures of four AlN/GaN DAs formed by 1 ML AlN with 1 ML GaN, 1 ML AlN with 4 MLs GaN, 4 MLs AlN with 1 ML GaN, and 4 MLs AlN with 4 MLs GaN, respectively. As shown in Fig. 3, tuning the thicknesses of the AlN barrier and GaN well remarkably changes the miniband structures of the AlN/GaN DA structure. By comparing the Fig. 3(a) to (b), increasing the GaN well thickness alone weakens the quantum confinement effect, which in turn reduce its effective energy gap from 4.76 eV to 3.97 eV. Besides, when the thickness of the AlN barrier increases, the quantum confinement will be enhanced resulting in the increased effective bandgap. Comparing Fig. 3(c) to (a), increasing the thickness of the AlN barrier from 1 ML to 4 MLs leads to the increase of effective bandgap from 4.76 eV to 5.24 eV. Same phenomenon can be also observed by comparing Fig. 3(d) to (b). In addition, both increasing the well and barrier thicknesses result in a weaker inter-well resonant coupling and narrower minibands.

The results presented in Fig. 3(a) to (d) indicate the importance of using an ultra-short-period superlattice structure for enhancing the inter-well resonant coupling, which will also increase the electron-hole wavefunction overlap for both emitters and absorbers applications. By tuning the thicknesses of the AlN barrier and GaN well layers, the energy band structure of the AlN/GaN DA can be precisely controlled to achieve different electronic and optoelectronic properties. Note that the superlattice effect in the DA structure will become negligible when the thickness of the barrier is much thicker than a threshold value (above ~ 7–9 MLs)^55,56^. Furthermore, the CH1 miniband is always lower than the HH1 miniband in the AlN/GaN DA when the thicknesses of AlN barrier and GaN well change from 1 ML to 4 MLs. In contrast to the dominant C-CH transition in high Al-content AlGaN QW^38^, the use of AlN / GaN DA structure enables the re-arrangement of the valence subbands resulting in dominant C-HH transitions for similar effective bandgaps.

**Figure 4:**
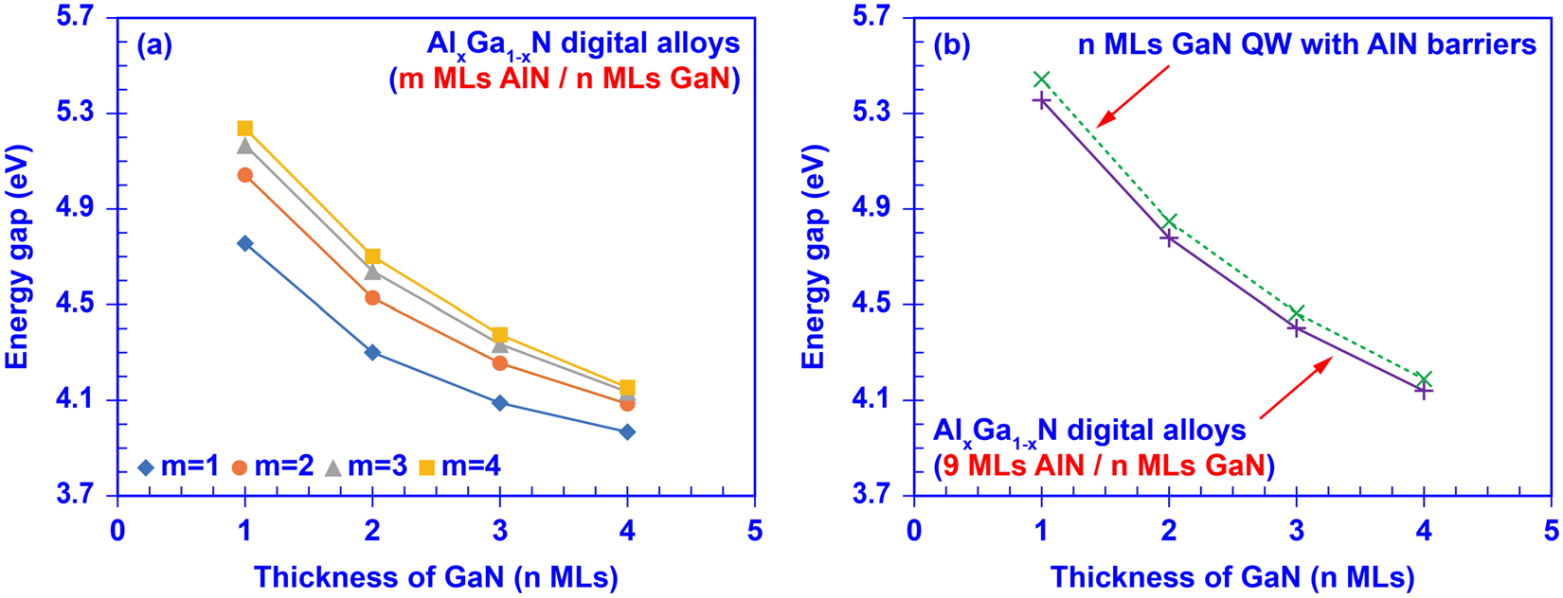
(**a**) Effective energy gaps of the AlN/GaN DAs formed by m MLs AlN barriers and n MLs GaN wells; (**b**) Effective energy gaps calculated by transfer matrix method for the AlN/GaN DAs with 9 MLs AlN barriers and n MLs GaN wells, and those calculated by self-consistent 6-band ***k*** **·** ***p*** method for the n MLs GaN QW with AlN barriers.

Figure 4 shows the range of tunability in the effective bandgap enabled from the usage of the AlN/GaN DA structure, which indicates the potential for accessing the entire mid-UV (λ ~ 310 nm) up to deep-UV (λ ~ 236 nm) spectral regime. The effective bandgap of the AlN/GaN DA is defined as the energy difference between the lowest confined state in C1 miniband and the highest confined state in HH1 miniband. Figure 4(a) shows the effective energy gap of the AlN/GaN DAs that is tuned from ~3.97 eV up to ~5.24 eV as a function of the thicknesses of the AlN barriers and GaN wells. As shown in Fig. 4(a), the increment of the effective energy gap becomes smaller for every additional ML of AlN barrier with a certain GaN well thickness. Once the AlN barrier in the AlN/GaN DA structure is thick enough, the increment of the effective bandgap will be relatively minimal indicating that its property is approaching that of the conventional MQW structure. Our calculation shows the strong capability of the AlN/GaN DA in tuning the effective energy gap via nano-structure engineering – varying the thicknesses of the ultra-thin AlN barrier and GaN well layers – for accessing the entire mid- and deep-UV spectral regimes.

Attributed to its negligible inter-well coupling, the properties of AlN/GaN DA with AlN barrier thickness above the threshold value (~7–9 MLs) will saturate at those presented in the conventional QW-like structure. This finding indicates that the effective bandgap for AlN/GaN DA structure with thick barrier layer ( > ~7–9 MLs) should approach that of the single GaN QW with AlN barrier structure. Figure 4(b) shows the comparison of the effective bandgaps for i) AlN / GaN DA structure with thick AlN barrier (9 MLs), and ii) single GaN QW with AlN barriers. This comparison was performed to confirm on the reliability of the transfer matrix method used in the DA calculation, where the ground-state transition energies of the single QW structure (n MLs GaN well with AlN barriers) were calculated by using self-consistent 6-band ***k*** **·** ***p*** method. In the single QW structures, the ground-state transition energies vary from 5.44 eV to 4.19 eV as the GaN well thickness increases from 1 ML to 4 MLs, which match well with the previously-reported values^62,63^. In the DA structure with 9 MLs AlN barrier, the effective bandgaps (~4.14 eV up to 5.36 eV) obtained with similar GaN well thicknesses also match very well with those in the single QW structure. The small (~1.2–1.6%) discrepancy in the results from the two numerical approaches provides a high degree of confidence on the results presented here.

**Figure 5:**
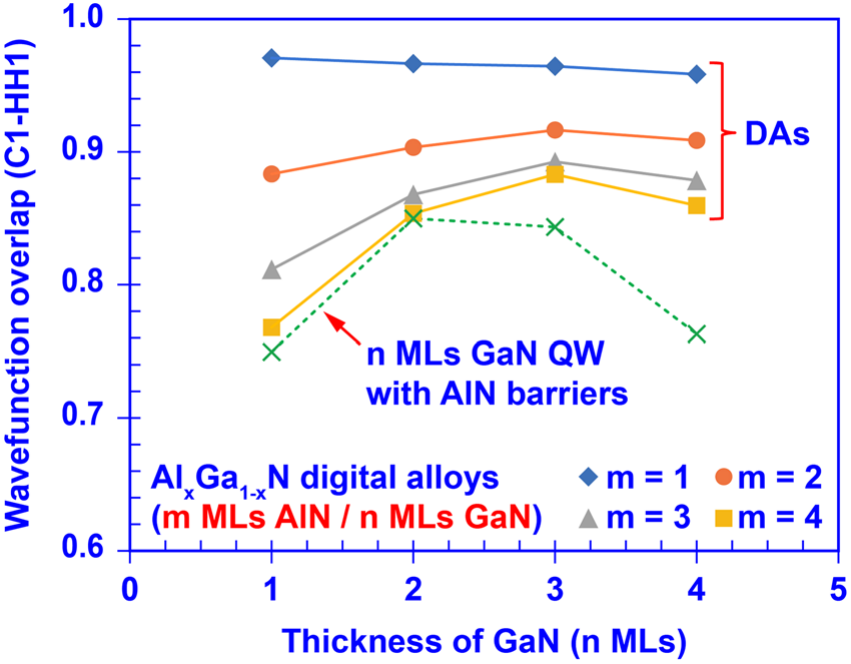
Ground state electron-hole wave function overlap of the AlN/GaN DA as function of the thickness of AlN barrier (m MLs) and thickness of GaN well (n MLs). The dash-line represents the electron-hole wave function overlap of the n MLs GaN QW with AlN barriers.

Attributed to its strong inter-well coupling, the use of ultra-short-period superlattice DA structure will have a profound impact on its electron-hole wavefunction overlap (Γ_e-h_). The large Γ_e-h_ in active region results in an increase in the optical matrix element^9,14^, which leads to larger spontaneous emission rate and optical absorption coefficient. As reported in previous work^56^, the ground-state electron-hole wavefunction overlap (Γ_C1-HH1_) in the InN/GaN DA structure can be enhanced up to >90% despite the existence of the built-in polarization field. Figure 5 shows the Γ_C1-HH1_ in the AlN/GaN DA structure as a function of various barrier and well thicknesses, and the comparison with the result from the single QW (with n MLs well thickness) is also presented. The use of single QW structure with thick GaN well thickness will result in relatively low overlap (~60%) arisen from the polarization-induced charge separation effect^9,44^. In the case of the ultra-thin single QW structure, an increased overlap up to ~72–80% can be obtained with relatively weak control of the effective bandgap. In the case of the DA structure (shown in Fig. 5), a relatively large Γ_C1-HH1_ of ~80% to ~97% can be obtained with large range of design parameters (GaN / AlN thicknesses). This also suggests that the use of optimized DA structure will provide tremendously high degree of control in the tuning of the effective bandgap by selecting the appropriate thickness ratios in the DA structures while maintaining large overlap above 85–95% in spite of the polarization-induced charge separation effect.

**Figure 6:**
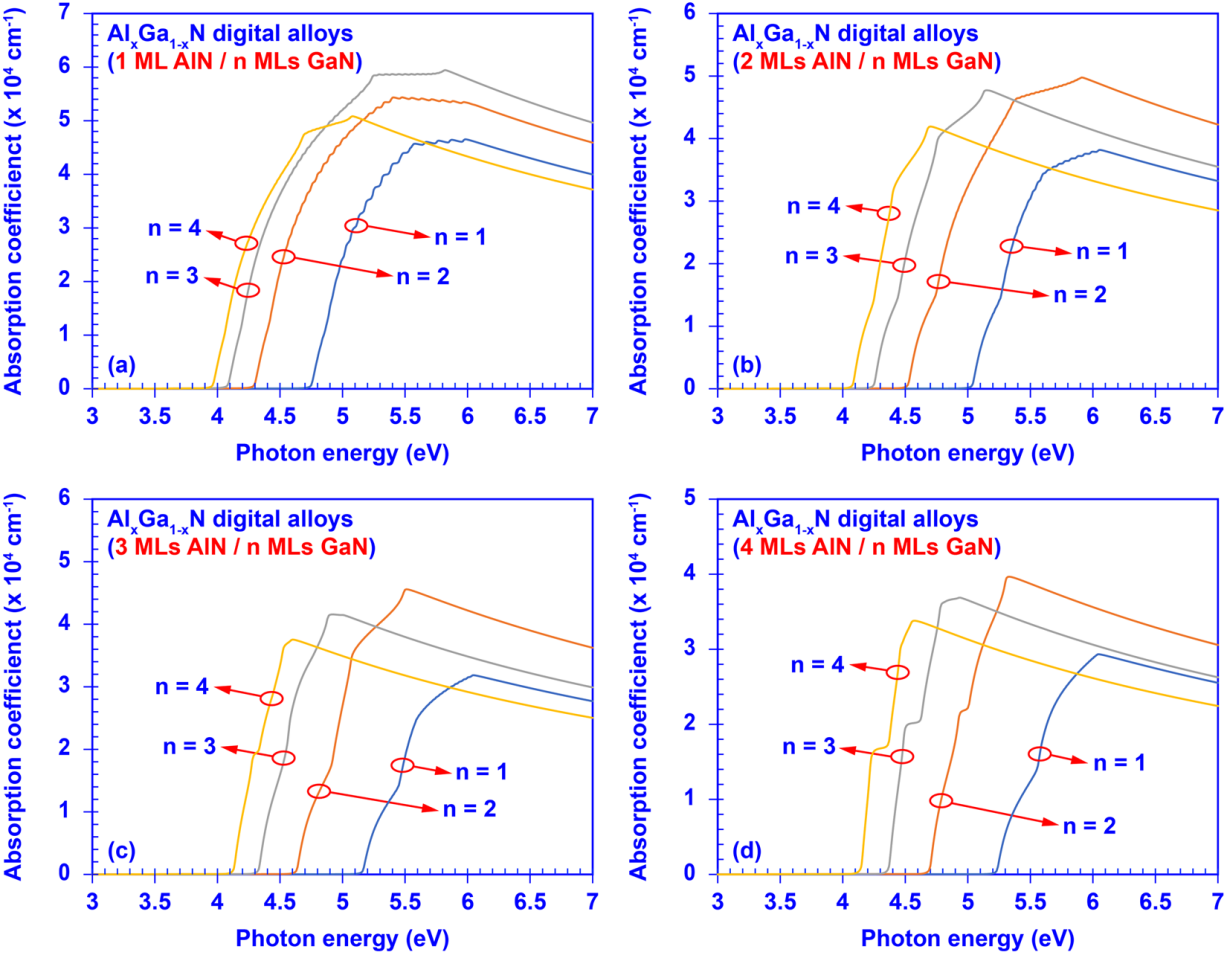
Absorption spectra of the 50-periods AlN/GaN digital alloys: set (**a**) 1 ML AlN with 1, 2, 3, 4 MLs GaN, respectively; set (**b**) 2 MLs AlN with 1, 2, 3, 4 MLs GaN, respectively; set (**c**) 3 MLs AlN with 1, 2, 3, 4 MLs GaN, respectively; and set (**d**) 4 MLs AlN with 1, 2, 3, 4 MLs GaN, respectively.

To provide a comprehensive understanding on the tunable optoelectronic properties, we calculated the absorption spectra of the AlN/GaN DAs with various barrier and well thicknesses. Figure 6 shows the calculated absorption spectra as function of the photon energy for sixteen 50-period AlN/GaN DAs with different barrier and well thicknesses. As shown in Fig. 6, these 16 AlN/GaN DAs are categorized into 4 groups, where the AlN barrier thickness is fixed at 1 ML, 2 MLs, 3 MLs, and 4 MLs, respectively. Then, the GaN well thickness is varied from 1 ML to 4 MLs in each group. In general, all the absorption spectra of the 50-periods AlN/GaN DAs are densely filled yielding the substantially broad coverage in UV spectral regime. As discussed previously, AlN/GaN DA with ultra-thin AlN barrier and GaN well layers results in a broad miniband structures. By adding up the periods of an AlN/GaN DA from a single QW to 50-periods DA, each miniband will start consisting of up to 50 non-degenerate confined states that split from the single quantum confined state in the QW. Eventually, all these confined states contribute to the optical transitions leading to the broadened absorption spectrum of the AlN/GaN DA structure. Specifically, the allowed transition between the ground state electrons and holes gives the minimum transition energy in the spectrum, while the allowed transition between the highest-state electrons and holes yields the maximum transition energy. For example, the AlN/GaN DA formed by 1 ML AlN and 3 MLs GaN has its interband absorption covering from ~4.1 eV to ~5.8 eV, which corresponds to a large bandwidth of ~90 nm in mid- and deep- UV regime. Moreover, the remarkably high Γ_e-h_ also contributes to the enhancement of the optical transition in the AlN/GaN DA active region. Note that the AlN/GaN heterostructure has been recognized to have promising radiation hardness in high power radiation^70,71^. Therefore, the wide coverage of the absorption spectrum of the AlN/GaN DA in mid- and deep-UV regime suggests its promising potential in high-energy photo-detection applications. In addition to the wide coverage of each absorption spectrum, the cut-off wavelengths of the absorption spectra shift from ~236 nm to ~312 nm when the thicknesses of the AlN barrier and GaN well vary from 1 ML to 4 MLs, which is corresponding to the tunable effective energy gaps. Our analysis shows that the optical transition of the AlN/GaN DA can be engineered by optimizing the structural design to achieve desired optoelectronic properties for mid- and deep-UV device applications.

## Conclusion

In summary, we present the comprehensive study on the electronic and optoelectronic properties of the AlN/GaN DA structure with ultra-thin ( ≤4 MLs) AlN barriers and GaN wells. Our analysis shows that the effective energy gap of the AlN/GaN DA structure can be engineered from ~3.97 eV (λ ~ 312 nm) up to ~5.24 eV (λ ~ 236 nm) by tuning the thicknesses of the AlN barrier and GaN well from 1 ML to 4 MLs. Our calculation also suggests that the valence band structure is properly rearranged in the AlN/GaN DA resulting in the desired HH band being the top valence subband, which provides the desired dominant TE-polarized emission for top-emitting emitters. Furthermore, our findings indicate that the charge separation issue induced by built-in polarization field in conventional AlGaN based QW can be overcome by applying ultra-thin AlN barrier and GaN well layers in the AlN/GaN DA. Compared to the GaN ultra-thin QW with AlN barriers structure, the AlN/GaN DA structure can achieve higher Γ_e-h_ (up to 97%) suggesting a possible solution for further enhancement of the internal quantum efficiency for high efficiency mid- and deep-UV top-emitting devices. In addition, the AlN/GaN DA also produces a wide absorption spectrum in mid- and deep-UV regime suggesting its promising potential in high-energy photo-detection applications. Our study shows the great potential of implementing the AlN/GaN DA with ultra-thin AlN barrier and GaN well layers as a promising active region design for high efficiency mid- and deep-UV top-emitting and top-absorbing device applications.
